# The Enhanced Performance of Oxide Thin-Film Transistors Fabricated by a Two-Step Deposition Pressure Process

**DOI:** 10.3390/nano14080690

**Published:** 2024-04-17

**Authors:** Mingjie Zhao, Jiahao Yan, Yaotian Wang, Qizhen Chen, Rongjun Cao, Hua Xu, Dong-Sing Wuu, Wan-Yu Wu, Feng-Min Lai, Shui-Yang Lien, Wenzhang Zhu

**Affiliations:** 1 Xiamen Key Laboratory of Development and Application for Advanced Semiconductor Coating Technology, The School of Opto-Electronic and Communication Engineering, Xiamen University of Technology, Xiamen 361024, China; 2015000077@xmut.edu.cn (M.Z.); 2122031310@s.xmut.edu.cn (J.Y.); 2122031278@s.xmut.edu.cn (Y.W.); chenqizhen@xmut.edu.cn (Q.C.); 2222031224@s.xmut.edu.cn (R.C.); wzzhu@xmut.edu.cn (W.Z.); 2 Guangzhou New Vision Opto-Electronic Technology Co., Ltd., Guangzhou 510640, China; xuhua@newvision-cn.com; 3 Department of Applied Materials and Optoelectronic Engineering, National Chi Nan University, Nantou 54561, Taiwan; dsw@ncnu.edu.tw; 4 Department of Materials Science and Engineering, National United University, Miaoli 360302, Taiwan; wywu@nuu.edu.tw; 5 Department of Biomedical Engineering, Da-Yeh University, Changhua 51591, Taiwan; fengmin@mail.dyu.edu.tw

**Keywords:** α-IGZO, thin-film transistor (TFT), HiPIMS, two-step deposition pressure, room-temperature fabrication

## Abstract

It is usually difficult to realize high mobility together with a low threshold voltage and good stability for amorphous oxide thin-film transistors (TFTs). In addition, a low fabrication temperature is preferred in terms of enhancing compatibility with the back end of line of the device. In this study, α-IGZO TFTs were prepared by high-power impulse magnetron sputtering (HiPIMS) at room temperature. The channel was prepared under a two-step deposition pressure process to modulate its electrical properties. X-ray photoelectron spectra revealed that the front-channel has a lower Ga content and a higher oxygen vacancy concentration than the back-channel. This process has the advantage of balancing high mobility and a low threshold voltage of the TFT when compared with a conventional homogeneous channel. It also has a simpler fabrication process than that of a dual active layer comprising heterogeneous materials. The HiPIMS process has the advantage of being a low temperature process for oxide TFTs.

## 1. Introduction

Large-area, active-matrix, organic, light-emitting diode displays depend on a thin-film transistor (TFT) back panel to provide a large driving current. A channel material that allows high mobility is necessary for the TFT to have a large output current at a specific size. However, traditional TFT techniques based on amorphous silicon and low temperature polysilicon cannot meet the requirements of high mobility and a good uniformity simultaneously [1]. An emerging amorphous oxide TFT technique represented by an amorphous indium-gallium-zinc oxide (α-IGZO) TFT provides a comprehensive solution for TFTs with high mobility and excellent uniformity [2].

At present, the mainstream method for preparing α-IGZO films in the flat panel display industry is RF magnetron sputtering (MS), which has a high film deposition rate and a large-area film deposition ability [3]. The film properties are somewhat inferior due to the existence of large number of defects when deposited by RFMS at room temperature. Generally, a post-annealing treatment at 250–450 °C is necessary to heal the defects, thereby improving the TFT performance [4,5,6,7]. However, the high temperature process would increase the thermal budget and reduce the compatibility with the back end of line (BEOL) of the device fabrication process and the thermal-sensitive flexible substrates. In addition, high mobility (*μ*) usually accompanies a high carrier concentration (*N*_c_) for an amorphous oxide semiconductor. Therefore, it is usually difficult to realize high mobility together with a low threshold voltage (*V*_th_) and good stability for amorphous oxide TFTs. A dual active layer (DAL) comprising heterogeneous materials has been raised as a solution to this problem [8,9,10,11,12]. However, it is complicated to deposit two different materials to form the DAL and it is difficult to control the heterointerface that has a significant influence on the electrical performance of the TFTs. Although a gradient channel modulated by the partial pressure of oxygen has been proposed in previous reports to simplify the process, the electrical properties of oxide semiconductors are generally very sensitive to the oxygen partial pressure (or oxygen flow rate) [13,14]. It usually leads to low control precision and poor controllability due to the minor amount of required reactive oxygen gas (of which the partial pressure is in the order of 10^−2^ Pa) and the accuracy limit of the gas mass flow controller. Therefore, a milder control parameter is needed.

In this study, α-IGZO films were prepared under a two-step deposition pressure process to modulate its electrical properties. The film was deposited by high-power impulse magnetron sputtering (HiPIMS) from a single target. The changing point of the two-step deposition pressures was adjusted to modulate the carrier population in the channel, thereby modulating the threshold voltage of the TFT while keeping high mobility. The fabrication process is simpler than that of a DAL comprising heterogeneous materials and the controllability of the deposition pressure is better than the oxygen partial pressure. In addition, the highly excited/ionized sputtered species during the HiPIMS process are expected to be beneficial for the low temperature fabrication of α-IGZO TFTs, which have better coverage on a high aspect ratio substrate.

## 2. Materials and Methods

**Figure 1:**
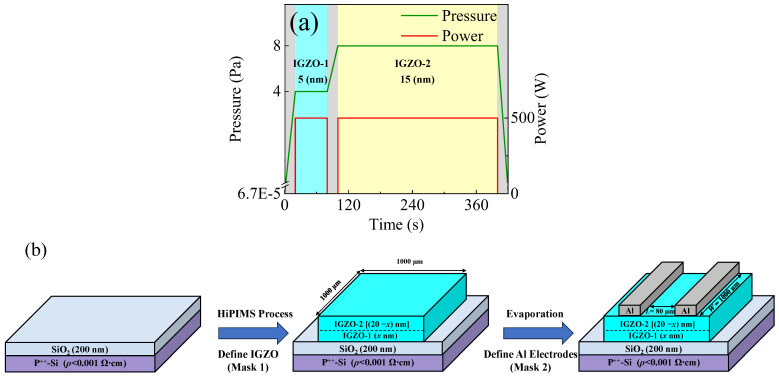
(**a**) Illustration of the two-step deposition pressure variation during the deposition of the IGZO channel (Ch.-III); and (**b**) the schematic description of the device fabrication process flow at each step.

IGZO films were deposited on silicon (Si) substrate from a ceramic target (purity = 99.99%; In_2_O_3_: Ga_2_O_3_: ZnO = 1:1:1; and dimensions: 12.7 cm × 30.5 cm) at room temperature using an in-line sputtering system (Ljuhv, SP-122I, Nantou, Taiwan). The base pressure of the chamber before the film deposition was pumped to 6.7 × 10^−5^ Pa. Argon gas with a purity of 99.999% and a flow rate of 40 sccm was used as the sputtering gas. A two-step deposition pressure (4 Pa/8 Pa) was applied during the film deposition process. The portions of the film deposited under 4 Pa and 8 Pa were denoted as IGZO-1 and IGZO-2, as illustrated in Figure 1a. The film thicknesses were measured by an α-step profiler (KLA-Tencor D-500, Hayward, CA, USA). The film deposition rates were obtained by dividing the film thicknesses with the corresponding deposition times. The film deposition rates at 4 Pa and 8 Pa were 5 nm/min and 3 nm/min, respectively. In general, increasing the deposition pressure can increase the deposition rate because a higher pressure chamber contains more ions, molecules, and free radicals. However, increasing the pressure can also decrease the deposition rate, as the mean free path of the sputtered atoms decreases, reducing the number of atoms that reach the substrate. The final variation of the deposition rate should be due to the result of the competition of the two mechanisms. The decrease of the deposition rate with an increasing deposition pressure suggested that the collision scattering dominated in influencing the film deposition rate. The thicknesses of the IGZO-1 and IGZO-2 were controlled by the deposition times according to the film deposition rates. The thickness ratio of the IGZO-1/IGZO-2 was adjusted by the changing point of the two-step deposition pressures. The total thickness of the IGZO channels were also verified by the α-step profiler. The power unit operated in a frequency-controlled mode. The impulse length, pulse-averaged power, and discharge voltage were set at 100 μs, 500 W, and 2000 V, respectively. Figure 1b shows a schematic description for the device fabrication process flow at each step. A heavily doped p-type (1 0 0) Si wafer (p^++^-Si, *ρ* < 0.001 Ω∙cm) was used as the substrate and the gate electrode. The silicon dioxide (SiO_2_, 200 nm) film grown by thermal oxidation served as the gate insulator. The α-IGZO film with a total thickness of 20 nm was deposited at room temperature on the SiO_2_ layer and patterned into islands by a metal mask. Then, an Al film (200 nm) was deposited by thermal evaporation and patterned by a metal mask to form the source/drain (S/D) electrodes. The width (*W*) and length (*L*) of the TFT were 1000 μm and 80 μm, respectively. The TFTs were fabricated at room temperature without post-annealing or other treatment and without a passivation layer.

The emissions from the plasma were analyzed using an intrusive optical emission spectrometer (OES) with the optics of the in-vacuum collimator set to avoid any spectra distortion due to film deposition on the detection optical lens in order to gather emissions in a wide wavelength range (200–1000 nm), including ultraviolent. The X-ray photoelectron spectra (XPS, Kratos, AXIS SUPRA+, Kyoto, Japan) were used to analyze the chemical states of the IGZO film with an IGZO-1/IGZO-2 thickness ratio of 5 nm/15 nm after the etching of the surface layer for different etching times by an Ar ion beam with 4 keV energy. At an etching rate of 0.31 nm/s, the etching times of 20 s, 40 s, 50 s, and 60 s correspond to different depths of 6.2 nm, 12.4 nm, 15.5 nm, and 18.6 nm from the film surface, respectively. The XPS spectra were calibrated by aligning the trace C 1s peak position at 284.8 eV. The electrical properties of the IGZO films grown on glass substrate were measured using a Hall effect test station (Ecopia, HMS5000, Anyang, Korea). The electrical performance of the TFTs were analyzed using a semiconductor analyzer (Keithley, 4200-SCS, Cleveland, OR, USA).

## 3. Results and Discussion

The constitution and electrical properties of the IGZO channels (Ch.-I–Ch.-V) are summarized in Table 1. The Ch.-V deposited at 4 Pa throughout the whole process has a high Hall mobility (*μ*_Hall_) of 13.7 cm^2^/V∙s and a high *N*_c_ of 5.7 × 10^18^/cm^3^. On the contrary, the Ch.-I deposited at 8 Pa throughout the whole process has a low *μ*_Hall_ of 3.9 cm^2^/V∙s and a low *N*_c_ of 2.1 × 10^16^/cm^3^. The variation of the carrier concentrations in Ch.-I and Ch.-V should be attributed to the variation in the Ga content and oxygen vacancy defect density indicated by the results of the OES spectra and XPS spectra that will be discussed below. The Ch.-III–Ch.-IV deposited at two-step deposition pressures have intermediate *μ*_Hall_ and *N*_c_ figures, both of which increase with the IGZO-1/IGZO-2 thickness ratio. The results suggested that the two-step deposition pressure process was able to modulate the electrical properties of the IGZO by adjusting the changing point of the deposition pressure. The deposition pressures are in the order of several pascals, which is much higher than the oxygen partial pressure in the order of 10^−2^ Pa, which was used to modulate the electrical properties of the gradient channel in previous reports. Therefore, it should have better controllability.

**Figure 2:**
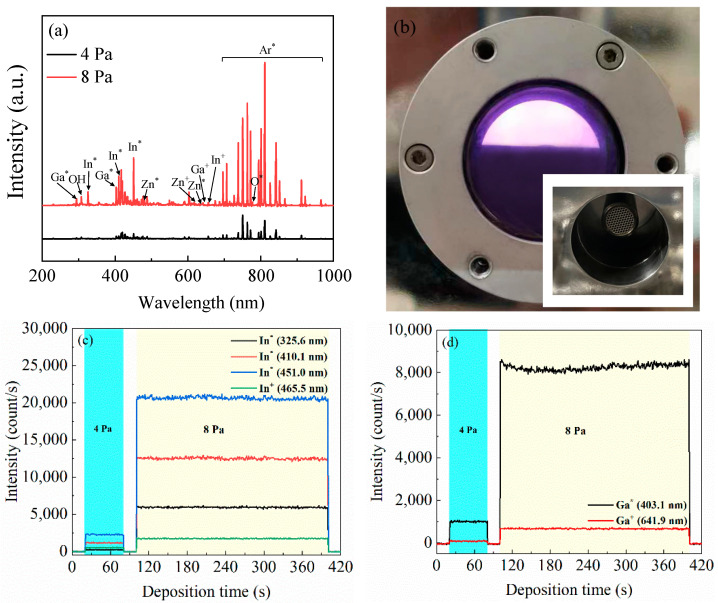

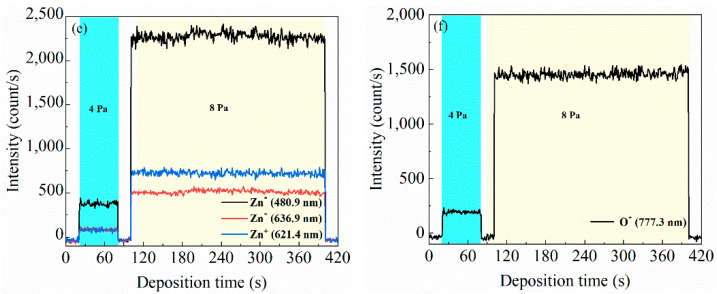
(**a**) The OES spectra of the plasma at 4 Pa and 8 Pa; (**b**) a photo image of the discharge plasma observed from the viewing port of the process chamber and the detection set viewed from inside the process chamber (the inset photograph); and (**c**–**f**) the variation of the OES intensity of each species with time during the deposition of Ch.-III. The superscript symbols *, and + denote neutral excited radicals and positive charged ions (cat-ions), respectively.

Figure 2a shows the OES spectra of the plasma at 4 Pa and 8 Pa. In addition to the emissions from the Ar radicals, the observed peaks were assigned to the emission from In* (centered at 325.6 nm, 410.1 nm, and 451.0 nm), In^+^ (centered at 465.5 nm), Ga* (centered at 403.1 nm), Ga^+^ (centered at 641.9 nm), Zn* (centered at 480.9 nm and 636.9 nm), Zn^+^ (centered at 621.4 nm), and O* (centered at 777.3 nm). The superscript symbols * and + denote neutral excited radicals and positive charged ions (cations), respectively. The emission intensities of the plasma species at 8 Pa are much stronger than those at 4 Pa due to the more frequent collision with the hot electrons and other species, leading to the more excited/ionized plasma species. Specifically, the oxidizing ability of the oxygen species with a higher excitation rate at a higher deposition pressure was expected to increase, leading to the suppression of the oxygen vacancy defect density, thereby lowering the carrier concentration in the film. Figure 2b presents a photo image of the discharge plasma observed from the viewing port of the process chamber and the detection set of the OES instrument viewed from inside the process chamber (the inset photograph). The intensity variations of each species over time during the deposition of Ch.-III are illustrated in Figure 2c–f. The results show that the OES intensities remain stable at each deposition pressure over the whole deposition process.

**Figure 3:**
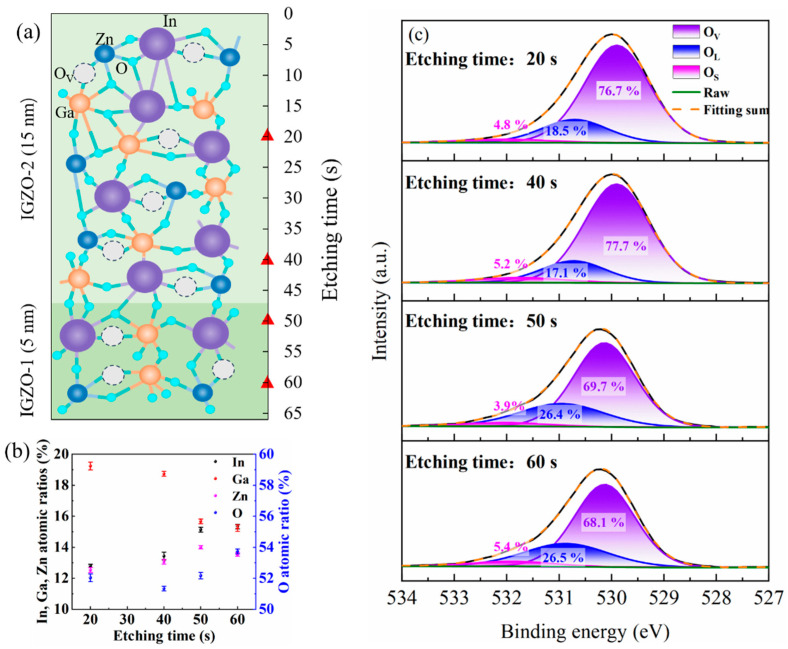
(**a**) The schematic diagram of the micro-structure of the α-IGZO channel prepared at two-step deposition pressures; (**b**) the relative atomic ratios of the IGZO film at different etching times; and (**c**) the high-resolution O 1s peak for the IGZO channel at different etching times (corresponding to different depths).

Figure 3a shows a schematic diagram of the micro-structure for the α-IGZO film prepared under two-step deposition pressures. Figure 3b shows the relative atomic ratios of the IGZO film at different etching times. The data were obtained from three samples prepared in one batch. The standard deviations of the concentrations of all species between samples are within 0.26%, indicating a good uniformity of the film in the same batch. For the etching times of 20 s and 40 s, the XPS signals are from the IGZO-2 region; for the etching times of 50 s and 60 s, the XPS signals are from the IGZO-1 region. The deviations of the concentrations of all species at different depths are small (with a maximum value of 0.7%) within the same region, except for that of the oxygen species at different depths in the IGZO-1 region, which has a significantly larger deviation of 1.57%. We noted that the depth corresponding to an etching time of 50 s is very close to the IGZO-1–IGZO-2 interface. The larger concentration deviation of the oxygen species is possibly due to the transition of the deposition pressure, which has a direct influence on the gaseous oxygen species with less influence on the metal species that are sputtered from the target. The results show that the In contents, Zn contents, and O contents are lower in the IGZO-2 region than those in the IGZO-1 region, while the Ga contents in the IGZO-2 region is higher than that in the IGZO-1 region. These represent the competition results of the sputtering yield, the self-sputtering yield, and the collision scattering of different plasma species [15,16,17]. Figure 3c shows the high-resolution O 1s peak for the IGZO channel at different etching times (corresponding to different depths). Each peak was fitted with three components centered around 530.0 eV, 531.0 eV, and 532.0 eV, corresponding to the oxygen atoms in the stoichiometric oxide lattice (O_L_), the oxygen atoms in the oxide lattice with oxygen vacancies (O_V_), and the surface chemisorbed oxygen groups or carbonate species on the film (O_S_). The area ratios of the Os peaks are randomly distributed, which is in accordance with the feature of their adventitious origins. The area ratios of O_V_/(O_V_+O_L_+O_S_) that reflect the relative concentration of oxygen vacancies in the IGZO-2 region are lower than those in the IGZO-1 region. One possible reason could be that the more oxygen radicals at the higher deposition pressure have a stronger passivation effect on the oxygen vacancies, which is in accordance with the analysis of the variation of the OES spectra for the oxygen species. The suppression of the oxygen vacancy density should lead to a lower carrier concentration in the film as oxygen vacancy defects have been identified as shallow donors in IGZO. As a result, the carrier concentration in the IGZO-2 is lower than that in the IGZO-2. Another possible reason could be that the higher Ga contents in the IGZO-2 (19.23 ± 0.25%, 18.73 ± 0.15%) than in the IGZO-1 (15.66 ± 0.15%, 15.23 ± 0.21%) suppressed the formation of oxygen vacancies, because Ga has a higher oxygen affinity than In and Zn. The specific values of the element contents and area ratios of O_V_/(O_V_+O_L_+O_S_) are listed in Table 2.

**Figure 4:**
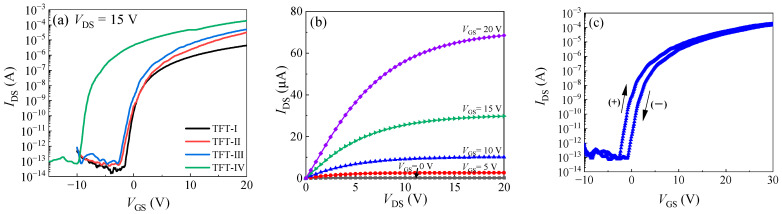
(**a**) The transfer curves for the TFTs with different channels; (**b**) the output curves; and (**c**) the dual sweep characteristics for TFT-III.

Figure 4a shows the transfer curves for the TFTs with different channels. The corresponding channel constitution and performance parameters of the TFTs are summarized in Table 3. For the channel prepared at a single deposition pressure (IGZO-1 or IGZO-2), it is difficult to realize high mobility together with a low threshold voltage (*V*_th_). For instance, TFT-I with the IGZO-2 channel layer has a low saturated field-effect mobility (*μ*_sat_) of 1.2 cm^2^/V∙s and a low *V*_th_ of 0.8 V, while TFT-V with the IGZO-1 channel layer has an unpractical negative *V*_th_ of −36.9 V due to the high *N*_c_ and carrier population [18]. In contrast, the TFTs with the IGZO channel prepared under two-step deposition pressures (TFT-II–TFT-IV) have the advantage of balancing high mobility and a low threshold voltage. The mobility increased with increasing the IGZO-1/IGZO-2 thickness ratio while keeping the *V*_th_ at around 0 V for TFT-II–TFT-III. However, the *V*_th_ exhibited a significant negative shift to −5.3 V for TFT-IV with a larger IGZO-1/IGZO-2 thickness ratio of 8 nm/12 nm. The optimal TFT (TFT-III) has a reasonable mobility of 4.4 cm^2^/V∙s, a small *SS* value of 0.34 V/decade, a low *V*_th_ of 0.4 V, and a low off current of 10^−13^ A. It combines a reasonable *μ*_sat_ and low *V*_th_ in contrast to those TFTs with a homogeneous channel. In addition, the off current for the TFTs with the IGZO channel prepared by the two-step deposition pressure process seems to be lower than the TFTs with a heterogeneous DAL channel (10^−11^ A), as reported in the literature. We speculate that the IGZO channels prepared by the two-step deposition pressure process have fewer defects than the heterogeneous DAL owing to the lack of a heterogeneous interface. Therefore, the off current was reduced owing to fewer de-trapping charges from the interfacial defects. Figure 4b shows the output curves of TFT-III. The curves exhibit a good linear feature at the linear region (*V*_DS_ < *V*_GS_), indicating a good contact property between the Al S/D electrodes and the IGZO channel. They also exhibit a good saturated feature at the saturation region (*V*_DS_ > *V*_GS_). Figure 4c shows the dual sweep characteristics of TFT- III. The forward- and backward-swept curves exhibited only a parallel shift without deterioration in the subthreshold characteristics, suggesting that there are no additional deep-level trap states created in the channel or the channel–insulator interface during the sweeping process. The hysteresis (Δ*V*_th_) between the forward- and backward-swept curves is 3.4 V. The hysteresis of the transfer curves should be ascribed to the trap of the carriers in the shallow trap states in the channel and at the channel–insulator interface, and the absorption/desorption of adventitious substances such as oxygen and moisture. Although the optimal saturated field-effect mobility is somewhat unsatisfied, this is likely to be due to the limit of the rough prototype device used, since the Hall mobility of 8.9 cm^2^/V∙s is reasonable for the α-IGZO film. Despite that, these results demonstrate that the two-step deposition pressure process is effective in controlling the electrical performance of the IGZO and the corresponding TFT performance.

**Figure 5:**
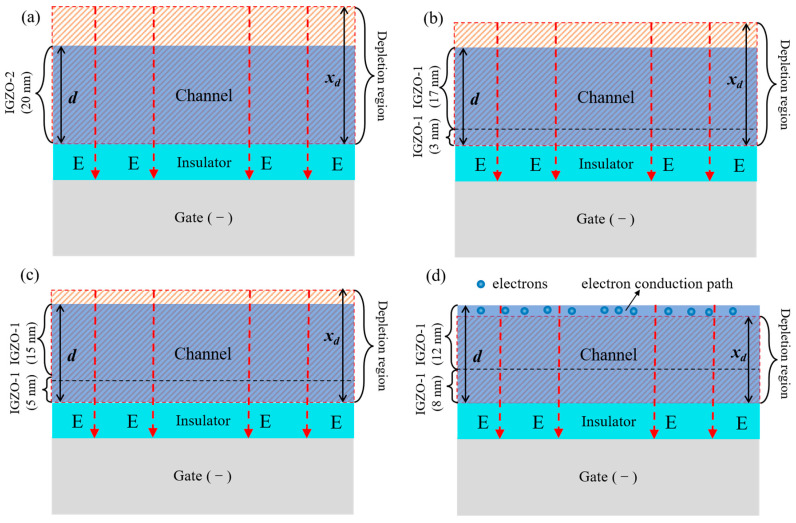
A mechanism for explaining the *V*_th_ shift results of the TFTs with the channel prepared at different deposition pressure conditions. (**a**–**c**) For TFT-I–TFT-III, the depletion width (*x*_d_) was thicker than the channel thickness (*d*), as a result, no conduction path was form in the channel. (**d**) For TFT-IV, the depletion width reduced to a value lower than the channel thickness; As a result, a conduction path remained at the back-channel region beyond the depletion region.

A mechanism was proposed to explain the *V*_th_ -shift results of the TFTs with the channel prepared under different conditions, as depicted in Figure 5. The average *N*_c_ in the channel was modulated by a different thickness combination of IGZO-1/IGZO-2. As a result of the negative bias exerted on the gate electrode, the free carriers were repelled from the channel–insulator interface by the electrical field, forming a depletion region in the channel. The depletion width (*x*_d_) is negatively related to the average *N*_c_ in the channel. For TFT-I–TFT-III, the depletion width was thicker than the channel thickness (*d*); thus, the channel can always be completely depleted to turn off the TFT, thereby keeping the *V*_th_ at around 0 V. For TFT-IV, the depletion width reduced to a value lower than the channel thickness due to the increase of *N*_c_; hence, a conduction path was formed at the back-channel region beyond the depletion region to form a current between the S/D electrodes, thereby leading to a significant negative shift in the *V*_th_.

The typical performance of the IGZO TFTs with a similar structure prepared by conventional DC or RF magnetron sputtering are summarized in Table 4 for comparison. When fabricated by DC or RF MS at room temperature, the TFTs exhibited an inferior performance [19,20] Generally, a post-annealing treatment at 250–450 °C is required to improve the performance [19,20,21,22,23,24]. In contrast, the IGZO TFTs prepared by HiPIMS have a small *SS* value, even if they were fabricated at room temperature without post-annealing or other treatment, implying that the defects in the IGZO channel were reduced. The highly excited/ionized species in the HiPIMS process seem to contribute to the improvement. First, they are beneficial for the synthesis of the metal oxide film at a low temperature, and, second, a dense film structure was expected with the bombardment of energetic species [25,26].

**Figure 6:**
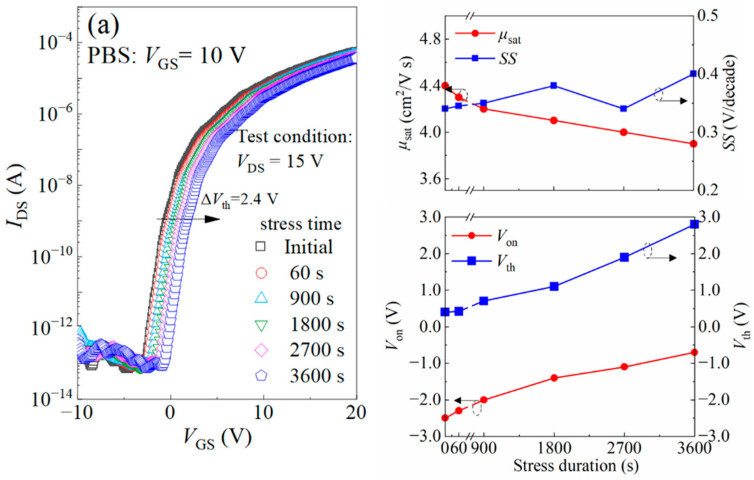

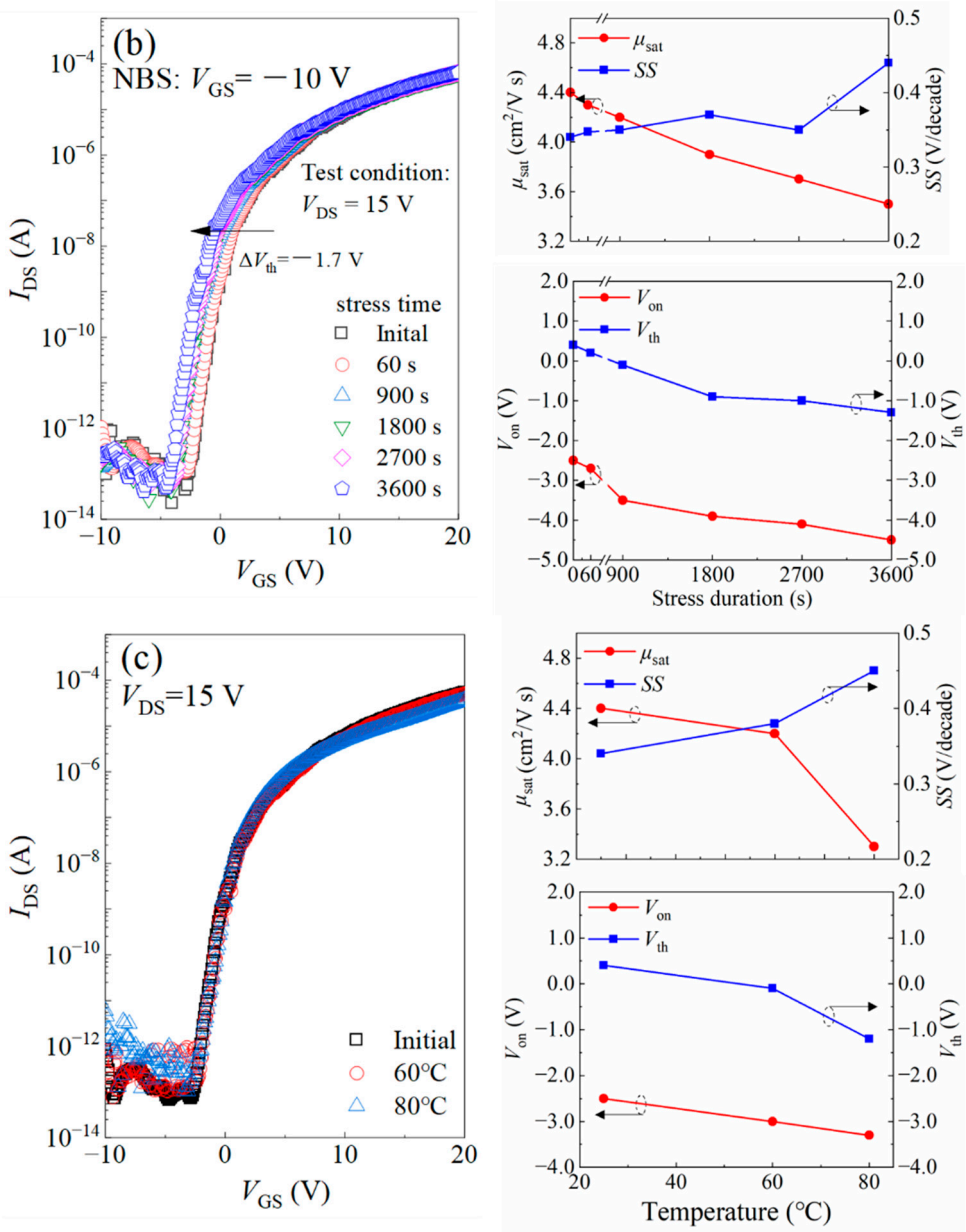
The development of the transfer curves and the corresponding key performance parameters of the TFT-III under (**a**) positive bias stress (PBS, *V*_GS_ = +10 V), (**b**) negative bias stress (NBS, *V*_GS_ = −10 V), and (**c**) under thermal bias with stress durations or testing temperatures.

The stabilities of TFT-III under bias stress or thermal stress were also evaluated. Figure 6a,b show the development of transfer curves and corresponding key performance parameters of the TFTs under positive bias stress (PBS, *V*_GS_ = +10 V) and negative bias stress (NBS, *V*_GS_ = −10 V) with the stress duration. After experiencing PBS for 3600 s, the *μ*_sat_ decreased from 4.4 cm^2^/V∙s to 3.9 cm^2^/V∙s; the *SS* value increased from 0.34 V/decade to 0.40 V/decade; and the shifts of *V*_on_ (Δ*V*_on_) and *V*_th_ (Δ*V*_th_) were 1.8 V and 2.4 V, respectively. After experiencing NBS for 3600 s, the *μ*_sat_ decreased from 4.4 cm^2^/V∙s to 3.5 cm^2^/V∙s; the *SS* value increased from 0.34 V/decade to 0.44 V/decade; and the Δ*V*_on_ and Δ*V*_th_ were −2 V and −1.7 V, respectively. The TFTs exhibited good bias stability, considering that they were not passivated. Figure 6c shows the transfer curves under different thermal stresses and the variation of the corresponding performance parameters with the testing temperature. Under thermal stress, the *μ*_sat_ exhibited only a slight decrease from 4.4 cm^2^/V∙s at the initial state to 4.2 cm^2^/V∙s and 3.3 cm^2^/V∙s at 60 °C and 80 °C, respectively; the *SS* value only increased slightly from 0.34 V/decade at the initial state to 0.38 V/decade and 0.45 V/decade at 60 °C and 80 °C, respectively; the shifts of *V*_on_ (Δ*V*_on_) were −3.0 V and −3.3 V at 60 °C and 80 °C, respectively; the shifts of *V*_th_ (Δ*V*_th_) were −0.1 V and −1.2 V, respectively; and the *I*_off_ increased from 10^−13^ A to 10^−12^ A. Overall, the variation of the TFT performance is not significant when taken to temperatures of 60 °C and 80 °C, which simulates what could be a real-world application.

## 4. Conclusions

IGZO TFTs were prepared by HiPIMS at room temperature. The HiPIMS process has the advantage of fabricating oxide TFTs at room temperature, thereby reduce the thermal budget and increasing the compatibility with the back end of line (BEOL) of the device integration and thermal-sensitive flexible substrates. The electrical properties of the channel were modulated by a two-step deposition pressure process. This process has the advantage of balancing high mobility and a low *V*_th_ of the amorphous oxide TFT. In addition, it has a simpler fabrication process and may have the potential advantage of reducing the off current in contrast to the heterogeneous DAL channel reported in the literature. The optimal TFTs exhibit a reasonable *μ*_sat_ of 4.4 cm^2^/V∙s, a small *SS* value of 0.34 V/decade, a low *V*_th_ of 0.4 V, and a high *I*_on_/*I*_off_ of 6.8 × 10^8^. They also exhibit good stability under PBS, NBS, and thermal stress.

## Figures and Tables

**Table 1:** The constitution and electrical properties of the IGZO channels.

Channel	IGZO-1 (nm)	IGZO-2 (nm)	*μ*_Hall_ (cm^2^/V∙s)	*N*_c_ (/cm^3^)
Ch.-I	0	20	3.9	2.1 × 10^16^
Ch.-II	3	17	5.5	8.8 × 10^16^
Ch.-III	5	15	8.9	1.7 × 10^17^
Ch.-IV	8	12	9.6	8.7 × 10^17^
Ch.-V	20	0	13.7	5.7 × 10^18^

**Table 2:** The relative atomic ratios of the In, Ga, Zn, and O elements and the relative oxygen vacancy concentration [O_V_/(O_V_+O_L_)] in IGZO films with different etching times.

Etching Time (s)	In (%)	Ga (%)	Zn (%)	O (%)	O_V_/(O_V_+O_L_+O_S_) (%)
20	12.80 ± 0.10	19.23 ± 0.25	12.53 ± 0.15	52.03 ± 0.25	18.5
40	13.43 ± 0.25	18.73 ± 0.15	13.07 ± 0.15	51.33 ± 0.15	17.1
50	15.13 ± 0.15	15.66 ± 0.15	14.00 ± 0.10	52.17 ± 0.21	26.4
60	15.33 ± 0.15	15.23 ± 0.21	13.53 ± 0.12	53.7 ± 0.15	26.5

**Table 3:** The performance of TFTs with different channels.

Device	Channel	*μ*_sat_ (cm^2^/V∙s)	*SS* (V/dec.)	*V*_th_ (V)	*I*_on_ (μA)	*I*_on_/*I*_off_
TFT-I	Ch.-I	1.2	0.28	0.8	4	8.8 × 10^7^
TFT-II	Ch.-II	2.9	0.46	0.7	30	4.8 × 10^8^
TFT-III	Ch.-III	4.4	0.34	0.4	51	6.4 × 10^8^
TFT-IV	Ch.-IV	6.0	0.24	−5.3	187	2.6 × 10^9^

**Table 4:** Comparison of the performance of the IGZO TFTs prepared by DC, RF magnetron sputtering, and HiPIMS.

Power Type	*μ*_sat_ (cm^2^/V∙s)	*SS* (V/dec.)	*V*_th_ (V)	*T* (°C)	Ref.
RF	0.4	0.88	4.8	RT	[19]
DC	4.3	0.61	2.5	RT	[20]
RF	8.3	0.23	3.5	250	[19]
RF	6.7	0.25	1.2	400	[22]
RF	10.3	0.18	−4.3	400	[23]
RF	13.1	0.50	1.7	450	[24]
DC	10.3	0.28	1.1	200	[20]
DC	11.2	0.26	0.2	290	[21]
HiPIMS	4.4	0.34	0.4	RT	This work

## Data Availability

Data are contained within the article.
